# Highly accelerated cardiac functional MRI in rodent hearts using compressed sensing and parallel imaging at 9.4T

**DOI:** 10.1186/1532-429X-14-S1-P65

**Published:** 2012-02-01

**Authors:** Tobias Wech, Craig A Lygate, Stefan Neubauer, Herbert Köstler, Jurgen E Schneider

**Affiliations:** 1 Institute of Radiology, University of Würzburg, Würzburg, Germany; 2 Cardiovascular Medicine, University of Oxford, Oxford, UK

## Summary

Parallel Imaging and Compressed Sensing have individually been shown to speed up cardiac functional MRI in mice and rats at ultra-high magnetic fields whilst providing accurate measurement of the physiologically relevant parameters. This study demonstrates that the acquisition time for cine-MRI in rodent hearts can be significantly reduced further by combining both techniques.

## Background

Parallel Imaging (PI) and Compressed Sensing (CS) are alternative approaches to reduce scan time in Magnetic Resonance Imaging (MRI). Specifically, PI (i.e. TGRAPPA) has recently been demonstrated to provide a three- to four-fold acceleration of cine-MRI in mice and rats (Schneider et al, MRM 2008 & 2011), while CS allowed for a three-fold reduction in scan time in normal mice and in a murine model of chronic myocardial infarction (Wech et al, JMRI 2011). In both cases, the increase in speed was achieved without impacting on the accuracy and reproducibility of cardiac functional parameters. We now hypothesized that PI and CS combined will allow to even further accelerate cine-MRI in rodent models of cardiovascular disease.

## Methods

All experiments were conducted on a horizontal, 9.4T MR system (Agilent) equipped with 8 receive-channels. Fully sampled cine-data were obtained in rats and mice, using a 2D multi-frame GE-sequence, and a 4-channel cardiac array and an 8-channel probehead, respectively. Matrix size (256 x 256) and temporal resolution (TR = 4.6ms) were identical for both cases. The fully acquired cine-data sets were retrospectively undersampled and subjected to two consecutive image reconstruction steps: first, a CS-algorithm reproducing k-t-SPARSE by Lustig et al (ISMRM 2006) was utilized to generate a moderately undersampled k-space. Subsequently, GRAPPA (Griswold et al, MRM 2002) was applied to obtain fully sampled cine-data.

## Results

**Figure 1:**
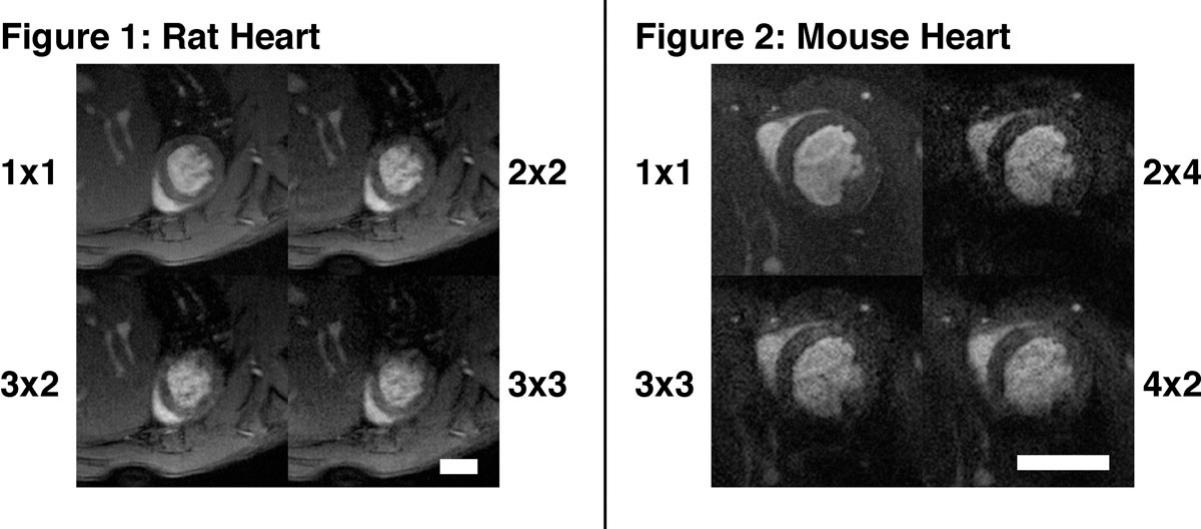


Figure 1 shows the end-diastolic frame of a mid-ventricular slice across a rat heart, comparing the fully sampled image (top left) to the corresponding frames with undersampling factors 4, 6 and 9. Figure 1 depicts equivalent data for a mouse heart, with undersampling factors 8 and 9. The scale bars indicate 5mm, and the acceleration factors are given as R_CS_ x R_PI_. Good image quality was obtained even for 9-fold undersampled data.

## Conclusions

Our data suggest that PI and CS combined will significantly reduce acquisition time down to the minute-range for cardiac functional MRI in rodents. Work is in progress to validate this technique quantitatively, and to determine the optimal combination of both acceleration techniques.

## Funding

This work was funded by the British Heart Foundation and the Elite Network of Bavaria.

